# Controlled Study of the Impact of a Virtual Program to Reduce Stigma Among University Students Toward People With Mental Disorders

**DOI:** 10.3389/fpsyt.2021.632252

**Published:** 2021-02-09

**Authors:** Matías E. Rodríguez-Rivas, Adolfo J. Cangas, Daniela Fuentes-Olavarría

**Affiliations:** ^1^Universidad del Desarrollo, Facultad de Medicina Clínica Alemana, Santiago, Chile; ^2^Department of Psychology, University of Almería, Almería, Spain

**Keywords:** stigma, undergraduate education, E-contact, standardized patient (SP), project based learning (PBL), multicomponent interventions, stigma reduction programme

## Abstract

Stigma toward mental disorders is one of today's most pressing global issues. The Covid-19 pandemic has exacerbated the barriers to social inclusion faced by individuals with mental disorders. Concurrently, stigma reduction interventions, especially those aimed at university students, have been more difficult to implement given social distancing and campus closures. As a result, alternative delivery for programs contributing to stigma reduction is required, such as online implementation. This paper reports the results of a controlled study focused on an online multi-component program on reducing stigma toward mental illness that included project-based learning, clinical simulations with standardized patients and E-Contact with real patients. A total of 40 undergraduate students from the Universidad del Desarrollo in Santiago, Chile, participated in the study. They were randomly divided between an intervention and control group. The intervention group participated in the online multi-component program, while the control group participated in an online educational program on cardiovascular health. We assessed the impact of the program by using the validated Spanish-language versions of the Attribution Questionnaire AQ-27 and the Questionnaire on Student Attitudes toward Schizophrenia with both groups, before and after the intervention. In addition, an *ad hoc* Likert scale ranging from 0 to 5 was used with the intervention group in order to assess the learning strategies implemented. Following the intervention, the participants belonging to the intervention group displayed significantly lower levels of stereotypes, perception of dangerousness, and global score toward people with schizophrenia (*p* < 0.001). In addition, participants presented lower levels of dangerousness-fear, avoidance, coercion, lack of solidarity, and global score (*p* < 0.001). The control group displayed no statistically significant differences in the level of stigma before and after the evaluation, for all of the items assessed. Finally, the overall assessment of each of the components of the program was highly positive. In conclusion, the study shows that online programs can contribute to reducing stigma toward mental disorders. The program assessed in this study had a positive impact on all the dimensions of stigma and all of the components of the program itself were positively evaluated by the participants.

## Introduction

Stigma toward mental disorders is one of today's most pressing global issues. (1), with important implications such as high levels of social exclusion (2), poor quality of life (3), and low social support (4), for those affected by them. Furthermore, there are barriers to access and continuity of education and employment (5, 6), and higher risks of dying by suicide (6) among this specific population.

Mental health professionals and medical students have been found to stigmatize psychiatric patients (7, 8), and 40% of people with a mental disorder report unfair treatment by health professionals such as medical doctors, psychiatrists, psychologists, and nurses (9, 10). Experiencing stigma in medical settings is a stressor that contributes, among others, to reducing patients' quality of life and to exacerbating inequities in health outcomes and access to healthcare (11–13). Furthermore, stigma is considered a major obstacle against processes of mental health recovery (14–16) and is a main determinant of the quality of care delivered by health professionals and medical students (17, 18).

At the international level, several strategies have been implemented within the general population, as well as medical students, to reduce stigma toward mental disorders (19), and the most successful ones have been those involving direct contact with patients (20, 21) as well as programs with strong educational components (16, 22–24). It has been shown that direct, person-to-person contact and activities where the participants and patients share thoughts and experiences around mental health are key to the success of these programs (25–27).

While these strategies have been implemented in different countries (28–30) and have led to decreasing stigma (31, 32) and promoting positive perceptions of psychiatric patients among students and professionals (33, 34), their implementation in virtual learning spaces is recent, particularly for university students (35). Several authors highlight the advantages of using multimedia resources, for instance audiovisual educational resources with standardized patients (36, 37), E-contact with psychiatric patients (38), and even the implementation of educational videogames (39, 40).

In the context of the SARS-CoV−2 pandemic, lockdowns and physical distancing recommendations, stigmatization and instances of discrimination against individuals with mental disorders have increased and have negatively impacted their well-being and quality of life (41). Additionally, the implementation of measures aimed at reducing the transmission of the virus have hampered the implementation of on-site interventions focused on the reduction of stigma toward psychiatric patients (42), and in that context, developing and implementing innovative and online alternatives is paramount.

Among such alternatives, for university students, active and interdisciplinary project-based learning, where students lead and develop a final project, has been proven useful (43, 44), as it promotes critical thinking, practical application of knowledge (45), and problem-solving skills (46). Its implementation in the training of health professionals on mental disorders is recent, however it has proven to effectively foster professional skills (47) and a comprehensive and inclusive understanding of the different social processes involved in mental health outcomes and care (48–50). As it allows students to reflect on their knowledge to implement their skills in a creative way (51), it is considered an innovative educational tool for the reduction of stigma toward mental health disorders and psychiatric patients.

In addition to project-based learning, the use of standardized patients has shown different benefits for skill training and e-learning in the field of education in psychiatry and mental health (52). Including it as part of educational intervention strategies has had a positive impact (53), particularly on skill and knowledge development, and for the promotion of a holistic understanding of mental disorders (54). However, working online rather than on-site with standardized patients in order to reduce stigma toward mental disorders is a recent, underexplored development in the field, which requires complementary educational interventions (37), along with the creation of original scripts focused on the story around experiencing the illness and the recovery process (55, 56).

Finally, E-contact, defined as “computer-mediated real-time interactions where members of different groups interact online” (57), has been used as a strategy for raising awareness and reducing prejudice among ideologically different groups (58, 59). However, its implementation in the field of stigma-reduction is new and innovative, and the existing evidence shows that it can reduce stigma toward the transgender population (60). With regards to the use of E-contact to reduce stigma toward mental disorders, to date, only one experimental study has been conducted, and has demonstrated that E-contact reduces anxiety, rage and stereotypes toward individuals with schizophrenia (38). In that sense, this confirms the relevance of including E-contact in interventions aimed at reducing stigma toward people with mental disorders, for instance through the use of synchronous videoconferencing (61).

The objective of this study is to demonstrate the effectiveness of a multi-component online intervention incorporating E-contact with mental healthcare patients, standardized patients, and a project-based learning program.

## Materials and Methods

### Participants

The participants were recruited within the Universidad del Desarrollo in Santiago, Chile, during “Innovation Week,” an event organized by the university, where students belonging to the first 3 years of undergraduate studies in different disciplines work on different innovative responses to local social issues.

The sample consisted of 40 university students in their first, second or third year, 32 of which studied health science degrees and the remaining 8 studied social science degrees. Eighty percent of the participants were women and 20% were men, all aged between 18 and 23 years old (X = 20.6; SD = 1.3). None of the participants had been trained in psychiatry.

The intervention group and control group were each made of 20 randomly allocated participants, with equal proportion of men and women, area of study, and no statistically significant difference with regards to age (*p* > 0.05).

### Instruments

#### Questionnaire on Student Attitudes Toward Schizophrenia (QSAS)

This instrument (62) aimed at evaluating the items of stereotyping toward schizophrenia and social distancing was developed for, and implemented with, high school students in Germany, during the World Program Against Stigma and Discrimination of the World Association of Psychiatry. The current study used the validated Spanish-language version developed by Navarro et al. (63), which possesses the appropriate psychometric properties, with a Cronbach's alpha of 0.95 for both evaluated factors.

#### Attribution Questionnaire (AQ−27)

This instrument (64) is used to quantify stigma toward people with mental disorders among the general population. It presents the participants with the case of a person diagnosed with schizophrenia and evaluates stereotyping and prejudice through a 9-point Likert scale. The current study used the Spanish-language, shorter version with 14 items, validated by Saavedra et al. (65), which possesses the appropriate psychometric properties for its four factors. (dangerousness-fear = 0.88, lack of solidarity = 0.837, coercion = 0.864, and avoidance = 0.758).

#### Learning Strategies Assessment Scale

This instrument was designed specifically for the current study. Participants assessed the integration of the different intervention strategies on a scale of 0 to 5, focusing on the perceived impact on empathy, understanding of recovery and social inclusion of people affected by severe mental disorders. In addition, the degree of recommendation of the program was assessed. The scale is available as Supplementary Material.

### Procedure

Prior to their participation in either group, the participants gave their written informed consent in accordance with the Declaration of Helsinki (66) and the Singapore statement on research integrity (67). The study was approved by the Ethics Committee of Universidad del Desarrollo, Santiago, Chile (protocol number: 2020–142). Once the participants were randomly allocated to the intervention and control groups, they all completed the AQ−27 and QSAS questionnaires described previously. These questionnaires were sent to the participants 15 min before the beginning of the study and were completed online. The experiment began once the participants had completed both questionnaires.

With respect to the intervention, the intervention group participated in the multi-component online program, which lasted 14 h equally distributed across two days. The program consisted of the three consecutive interventions described below:

- Two sessions including a simulation with standardized patients with emotional, substance use and anxiety disorders and an education workshop. These common mental disorders are psychiatric comorbidities and are highly prevalent among patients with schizophrenia (68, 69). An online presentation was carried out by two trained actors focused on the main characteristics of these disorders. Previous to the presentation, the research team had designed original scripts based on the recovery process of the patient and their family environment. During every simulation, dynamic interactions took place between the trained actors and the students, who asked them, for instance, about their life-experience, how they were feeling, etc. Additionally, an educational workshop was carried out for each simulation, in order to discuss the essential aspects of the mental disorders presented during the simulation. Each session lasted 2.5 h.
- E-contact activity with an adult diagnosed with schizophrenia, who discussed and analyzed their recovery process with the students, including the role of the healthcare system, the community and their family. This activity lasted 3.5 h.
- Project-Based Learning. During the two days of the program, activities aimed at developing an intervention around the reduction of stigma and the promotion of social inclusion for people with mental disorders, designed and led by the students were implemented. At the end of the second day, the students had the opportunity to present, in a webinar, the interventions they had developed throughout the course of the program, to the other participants and to the members of the evaluation jury, which included the patient with whom the E-contact activity was carried out. This allowed the participants to receive feedback from an individual who had experienced a severe mental disorder. The presentations were carried out in groups, and the interventions introduced were related to the promotion of social inclusion and the rights of people affected by mental disorders in the academic and local community, with emphasis on raising awareness around stigma and education on mental health. This activity had a duration of 5.5 h, distributed between the development of the initiative, tutoring from the teaching team and the final presentations. An example of one of the projects carried out by the students is available as Supplementary Material.

The control group participated in an online educational program on cardiovascular health of the same duration.

Finally, at the end of both days, each group answered the AQ-27 and QSAS questionnaires. Additionally, the intervention group completed the Learning Strategies Assessment Scale.

**Figure 1:**
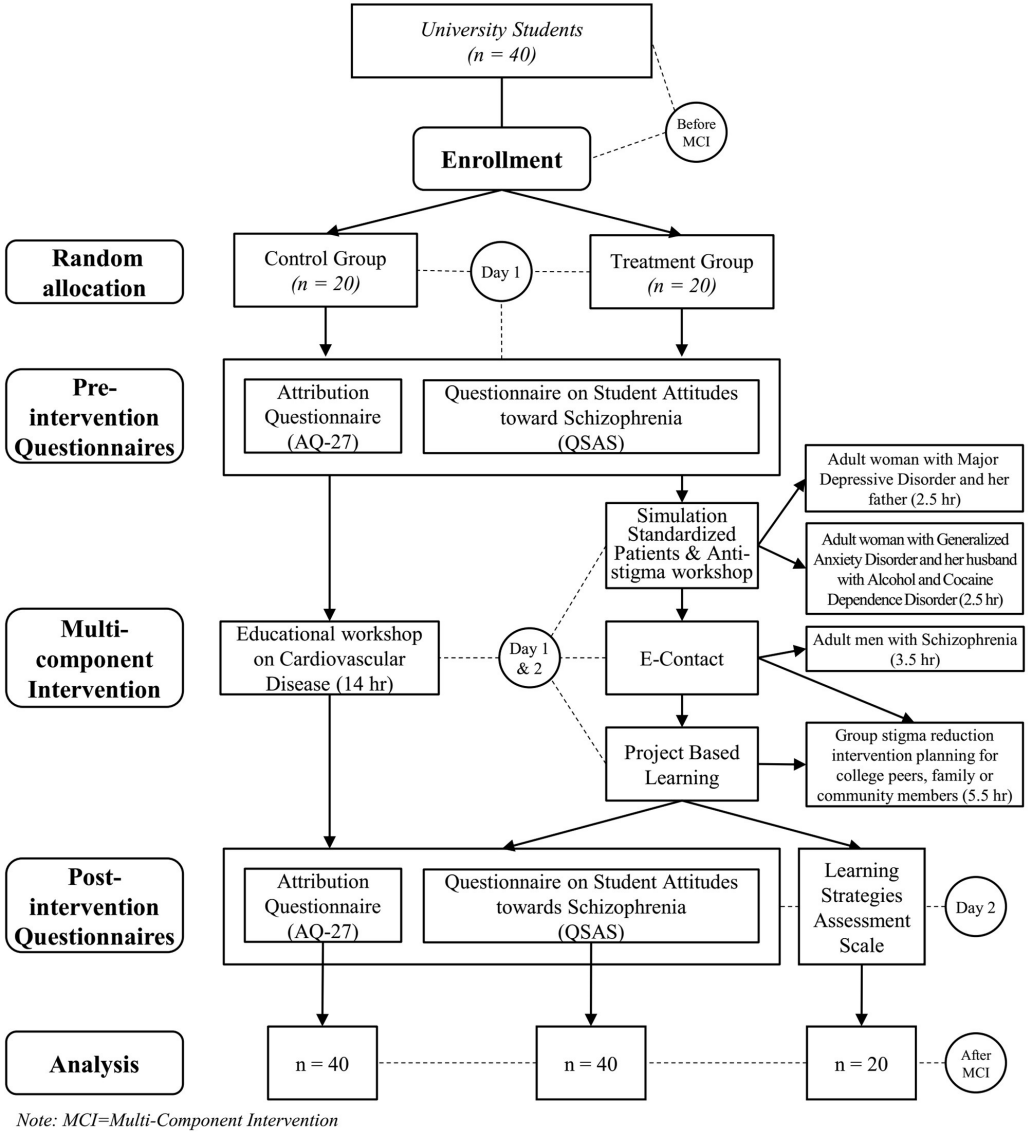
Participant assignment procedure to the multicomponent intervention [MCI].

Figure 1 summarizes the whole procedure of the study.

### Statistical Analysis

Student's *t*-test for independent samples was used to assess whether there were statistically significant differences between the pre- and post-intervention measures in both groups. Additionally, Cohen's d was used to determine the effect size of the intervention.

The pre- and post-measures of each group were subsequently analyzed with the use of Student's t for related samples, which was complemented with the measure of the effect size of the intervention through Cohen's d. Finally, descriptive statistics were used for the analysis of the global assessment of each one of the intervention strategies. We used SPSS 22.0 software to carry out the statistical analysis.

## Results

As Table 1 shows, there is no statistical difference between the intervention and control groups prior to the intervention for any of the items of either instruments. However, statistically significant differences are observed for the group means in the post-intervention measures for both groups for all of the analyzed variables. Using Cohen's d effect size allowed us to determine a great effect size for all of the dimensions evaluated for both instruments, except for the stereotypes indicator, which displays a more conservative effect size.

**Table 1:** Student's *t*-test for independent samples of pre-test and post-test differences between the intervention group and control group.

	**Pre-test**			**Post-test**		
**Variable**	***t***	***p***	***d***	***t***	***p***	***d***
**QSAS**						
Dangerousness	0.48	0.63	0.16	4.88	0.000	1.45
Stereotypes	−0.45	0.65	−0.16	2.10	0.04	0.76
Total	0.09	0.92	0.03	4.24	0.000	1.40
**AQ−27**						
Dangerousness-Fear	0.27	0.78	0.08	9.76	0.000	2.27
Avoidance	−1.83	0.08	−0.47	6.29	0.000	1.81
Coercion	−0.69	0.49	−0.26	6.88	0.000	2.25
Lack of Solidarity	0.98	0.33	0.31	3.27	0.004	1.00
Total	−0.38	0.70	−0.11	11.61	0.000	2.64

*QSAS, Questionnaire on Student Attitudes toward Schizophrenia, Adapted from Schulze et al. (62)*.

*AQ−27 = Attribution Questionnaire, Adapted from Corrigan et al. (64)*.

Table 2 shows the average means and standard deviations for each variable for both instruments corresponding to the intervention and control groups for each phase of the study. The analysis of the post-intervention scores of the control group shows that there is no statistically significant difference for any of the variables studied in either instruments. However, statistically significant differences are observed in the same analysis of the scores for the intervention group, for all of the scores of the questionnaires in relation to stigma in every proportion. Regarding the effect size, the program had a positive impact on stigma reduction as reflected on each one of the analyzed items and the global score of both questionnaires.

**Table 2:** Means and standard deviations of pre-test, post-test, and Student's *t*-test for related samples of post-test–pre-test differences in the study variables for the intervention group and control group.

	**Intervention group**							**Control group**						
	**Pre-test**		**Post-test**		**Pre-post**			**Pre-test**		**Post-test**		**Pre-post**										
**Variable**	***M***	***SD***	***M***	***SD***	***t***	***p***	***d***	***M***	***SD***	***M***	***SD***	***t***	***p***	***d***
**QSAS**														
Dangerousness	3.20	2.37	1.15	1.53	3.00	0.007	1.02	3.55	1.70	3.45	1.63	0.80	0.42	0.05
Stereotypes	3.05	1.50	1.90	1.25	2.52	0.02	0.83	2.8	1.47	2.95	1.46	−1.14	0.26	−0.10
Total	6.25	3.43	3.05	2.16	3.24	0.004	1.11	6.35	2.64	6.40	2.60	−0.43	0.66	−0.01
**AQ−27**														
Dangerousness-Fear	17.4	7.54	7.5	2.81	6.41	0.000	1.73	18.05	7.2	18.3	6.08	−0.56	0.58	−0.03
Avoidance	11.85	4.34	4.2	1.67	8.66	0.000	2.32	9.60	5.09	10.05	4.23	−1.33	0.19	−0.09
Coercion	10.9	4.62	4.65	1.87	7.70	0.000	1.77	9.85	3.16	10.20	2.93	−1.27	0.21	−0.11
Lack of Solidarity	7.05	3.10	5.00	1.52	3.66	0.002	0.83	8.15	3.88	7.7	3.46	2.01	0.05	0.12
Total	47.2	14.22	21.35	6.52	9.90	0.000	2.33	45.65	13.57	46.25	11.58	−0.88	0.38	−0.04

*QSAS, Questionnaire on Student Attitudes toward Schizophrenia, Adapted from Schulze et al. (62)*.

*AQ-27, Attribution Questionnaire, Adapted from Corrigan et al. (64)*.

The assessment given by the participants on the intervention was measured with a Likert scale ranging from 0 to 5 points. As Table 3 shows, 95% (*n* = 19) of the students assessed at the top level the usefulness of the program in understanding the recovery process and promoting social inclusion of patients with severe mental disorder, while 100% (*n* = 20) of them rated at the top level the promotion of empathy. Additionally, 95% (*n* = 19) stated they would recommend this program to a peer. There was no statistically significant differences between the three interventions and their integration. Finally, positive and uniform comments were presented, also in conjunction with the following representative verbatim quotes: “The activities we carried out, helped me to have a better view of mental illness, and the use of simulated patients was useful in the experience and learning”; “I really liked the simulations and the E-Contact, it makes it much more didactic to learn and understand about mental illness” and “was a wonderful experience, it would be a huge challenge to do it on-site.”

**Table 3:** Intervention group participants who rate the integration of learning strategies at the top level (*n* = 20).

	**Integration of the 3 types of learning strategies**	
**Variable**	***n***	***%***
Understanding of recovery of SMD	19	95
Promotion of empathy toward SMD	20	100
Promotion of social inclusion of SMD patients	19	95
Would recommend the program to a peer	19	95

*SMD, Severe Mental Disorder*.

*Top level rate, Top score of Learning Strategies Assessment Scale (5 points)*.

## Discussion

Stigma toward mental disorders is a pressing issue, considering, on the one hand, its impact on the wellbeing and quality of life of individuals with mental disorders (14–19), and on the other hand, that it is pervasive among the general population, health professionals and university students (7–10, 70–72).

At present, and to the best of our knowledge, there is no multi-component program focused on stigma toward mental disorders entirely carried out online. For this reason, as well as the lockdowns and social distancing measures implemented as a result of the SARS-CoV−2 pandemic, this study focuses on the implementation of an online program.

The results show that the intervention had a positive impact on the reduction of stigma among the intervention group. These results may be explained by the intensity of the program, the integration of multiple components, the focus given to the recovery process and the implementation of direct E-contact between mental health patients and students, all of which have been demonstrated to be key points for the implementation of a successful stigma-reduction program (54–58, 73–77). Additionally, further studies comparing the effectiveness of implementing the multicomponent program on-site rather than online, are necessary. Other studies have shown that comparable online interventions have an impact similar to those implemented on-site (27, 78).

Considering that the intervention was intensive, taking into account the number of activities carried out in a short amount of time, further qualitative studies will explore the experience of the participants and the lessons learned from the implementation of the key components, in order to adapt and adjust the program and make it easier to replicate.

It is important to emphasize that the assessment that the students made of the program was positive with respect to online implementation for each of its components, and the components focused on the promotion of empathy, social inclusion and understanding of the recovery process of severe mental disorders scored the highest, especially when integrated together. This suggests that the participants enjoyed the experience, would recommend it to peers, bringing evidence on the advantages of interventions that take into account the motivation of university students toward learning and goal-achievement, which is in turn one of the main challenges of digital education (79, 80).

In that sense, the educational intervention methods used in the current study are supported by robust theoretical and empirical evidence, and are especially adequate for online implementation, which is key in the context of the SARS-CoV-2 pandemic and the restriction of on-site interventions (36–40, 53–56). This also represents an opportunity for future integration of similar programs in university curricula, as they offer the possibility to reach remote areas, to establish contact between people with different experiences around mental disorders and between patient communities through E-contact and to generate student-led projects. Furthermore, the online modality of these programs promotes collaborative work between individuals and institutions across different countries, contributing, on the one hand, to foster cultural diversity, and on the other hand, to develop international education networks (81, 82).

In conclusion, the design and implementation of this type of interventions and educational spaces focused on diminishing stigma toward psychiatric patients contribute to normalizing these experiences among the population, by openly sharing challenges around mental health and promoting effective help-seeking and the delivery of adequate treatment to those who need it (83, 84). The study shows that online programs can promote new types of interventions aimed at reducing stigma not only in a context of mandatory social-distancing and lockdowns due to the SARS-CoV-2 pandemic, but also complementing existing on-site programs.

However, the study presents several limitations. First, the sample is relatively small, comes from one specific institution and is not representative of the general population, which may undermine the generalization of the results and limit the interpretation of the effect size. Second, the phases of the intervention were not experimentally evaluated in an independent way and the measure of stigma was only evaluated for schizophrenia, making it necessary to include other prevalent mental disorders in future research. Third, specific characteristics of the participants and possible confounding factors, which may influence the results, such as their socio-cultural background and their level of knowledge, desire to work in psychiatry and closeness to mental disorders were not evaluated. Finally, the long duration of the program may limit its replication, presenting several logistical challenges in its implementation through virtual platforms, specifically when considering factors such as student motivation and the exhaustion experienced in this regard.

## Data Availability Statement

The datasets presented in this study can be found in online repositories. This data can be found here: https://data.mendeley.com/datasets/p5d39f3kp6/1.

## Ethics Statement

The studies involving human participants were reviewed and approved by Ethics Committee of Universidad del Desarrollo, Santiago de Chile (protocol number: 2020–142). The patients/participants provided their written informed consent to participate in this study.

## Author Contributions

MR-R, AJC, and DF-O contributed to the conception, coordination, and design of the work. MR-R and DF-O contributed to the implementation of the program. MR-R and AJC performed the statistical analysis. All authors contributed to the article and approved the submitted version.

## Conflict of Interest

The authors declare that the research was conducted in the absence of any commercial or financial relationships that could be construed as a potential conflict of interest.
