# Supplementary material for: Controlled Study of the Impact of a Virtual Program to Reduce Stigma Among University Students Toward People With Mental Disorders
Source: Front Psychiatry. 2021 Feb 9;12:632252. doi: 10.3389/fpsyt.2021.632252 (PMC7900522; doi:10.3389/fpsyt.2021.632252)
Supplement: Supplementary file 1 [file Data_Sheet_1.DOCX]

Supplementary Material

# Supplementary Figure:

***Example of a project designed by students, in which they explain how they would carry out a stigma reduction intervention towards mental illness***


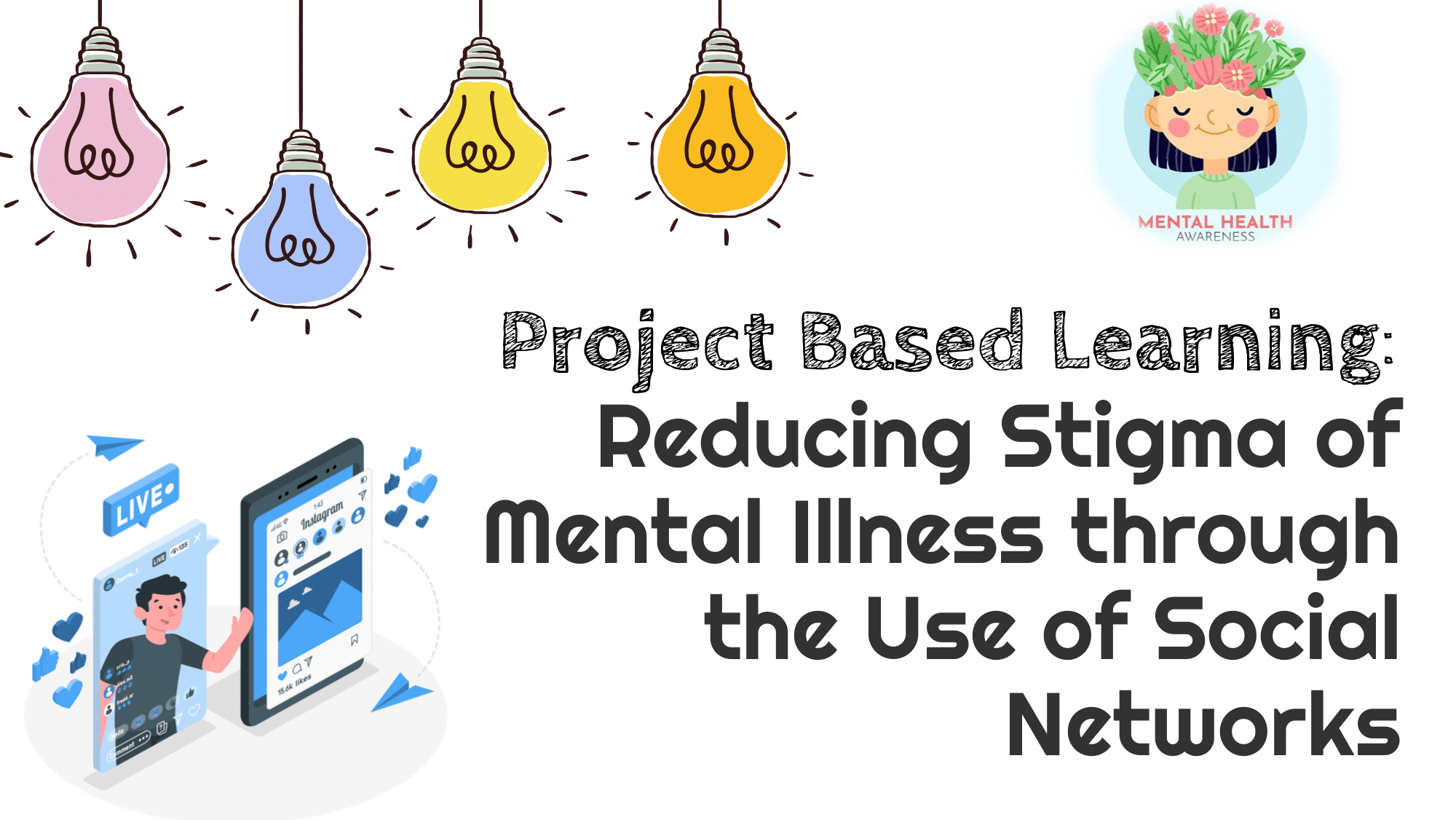


**A**


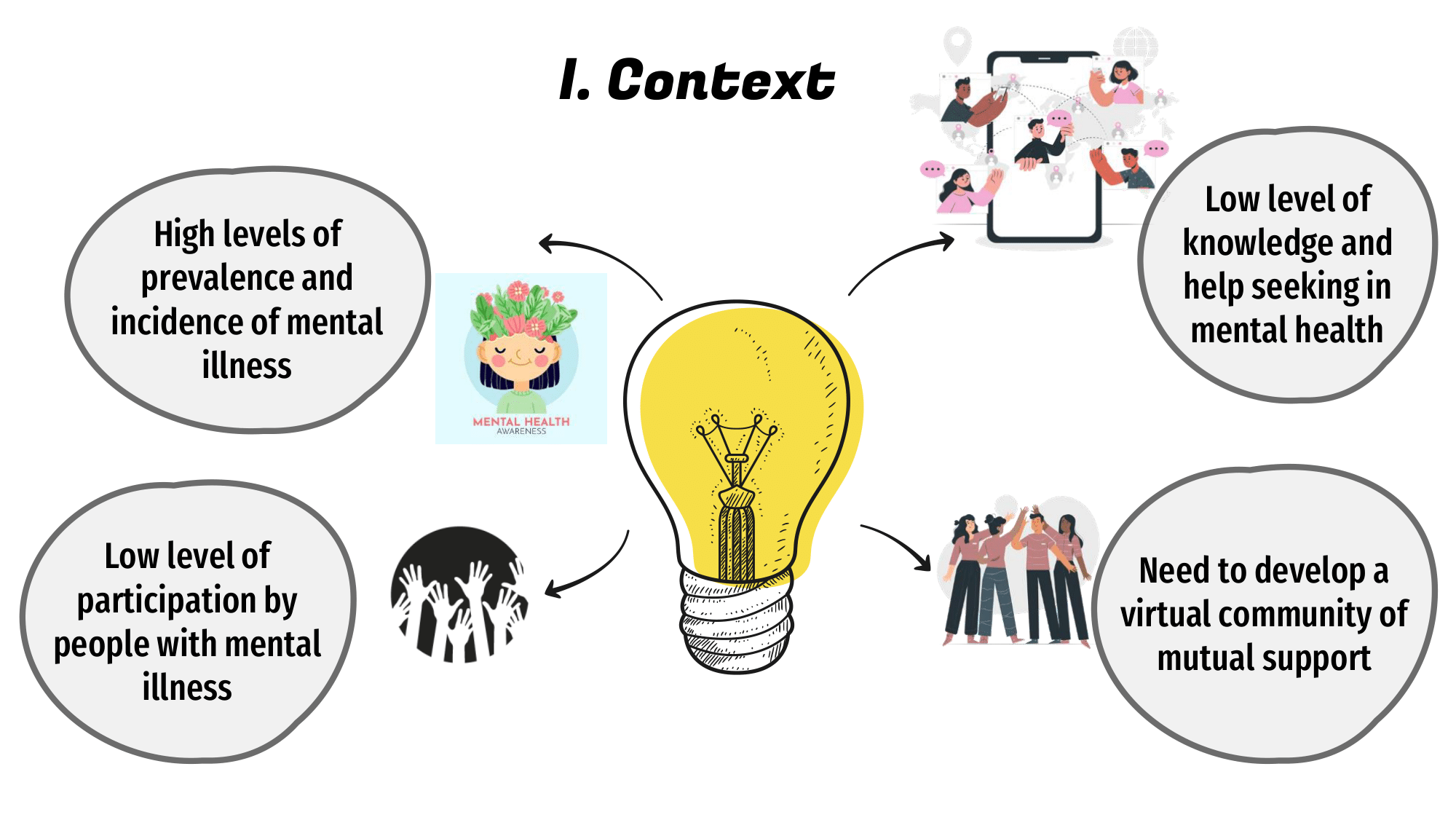


**B**


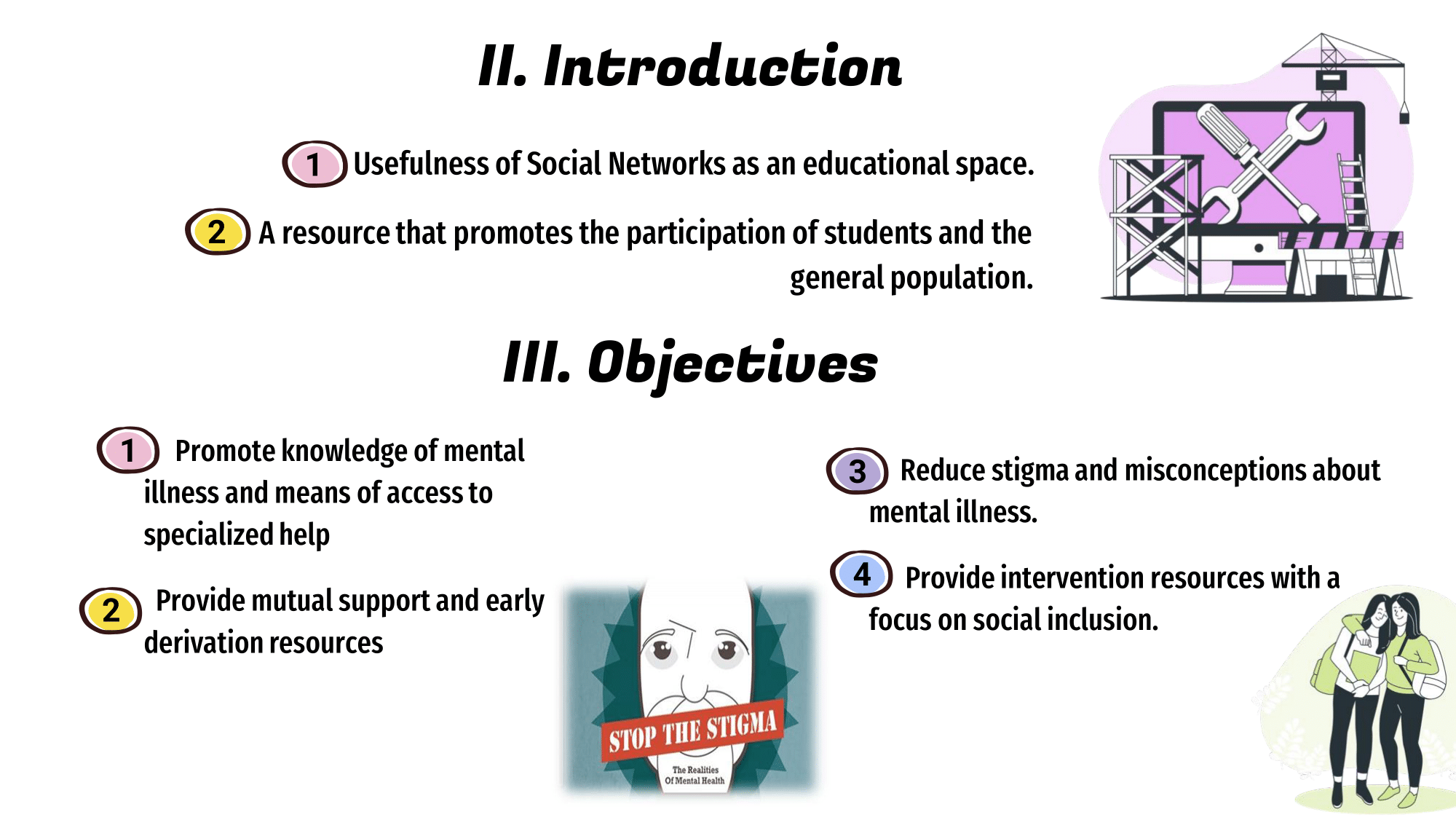


**C**


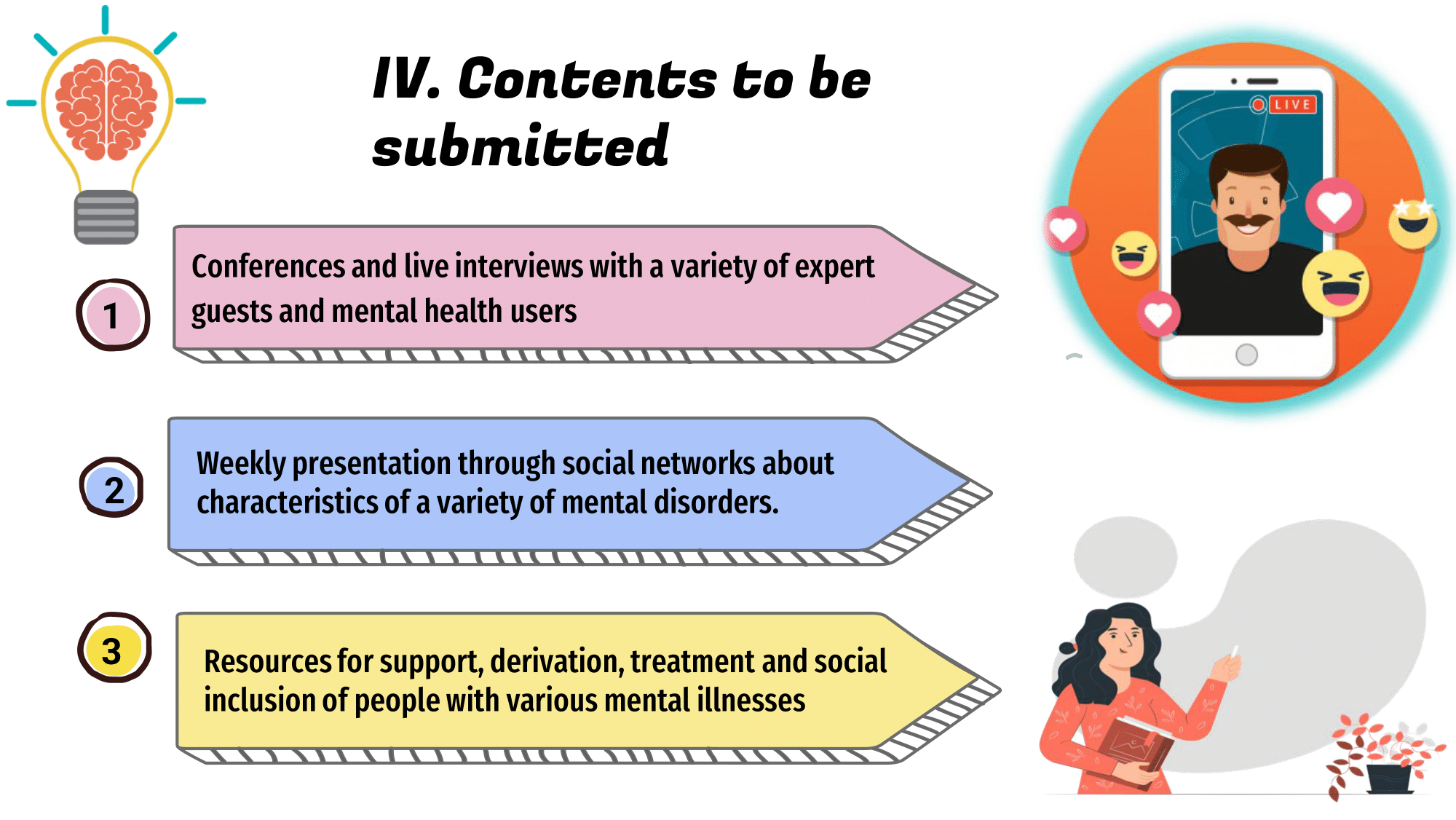


**D**


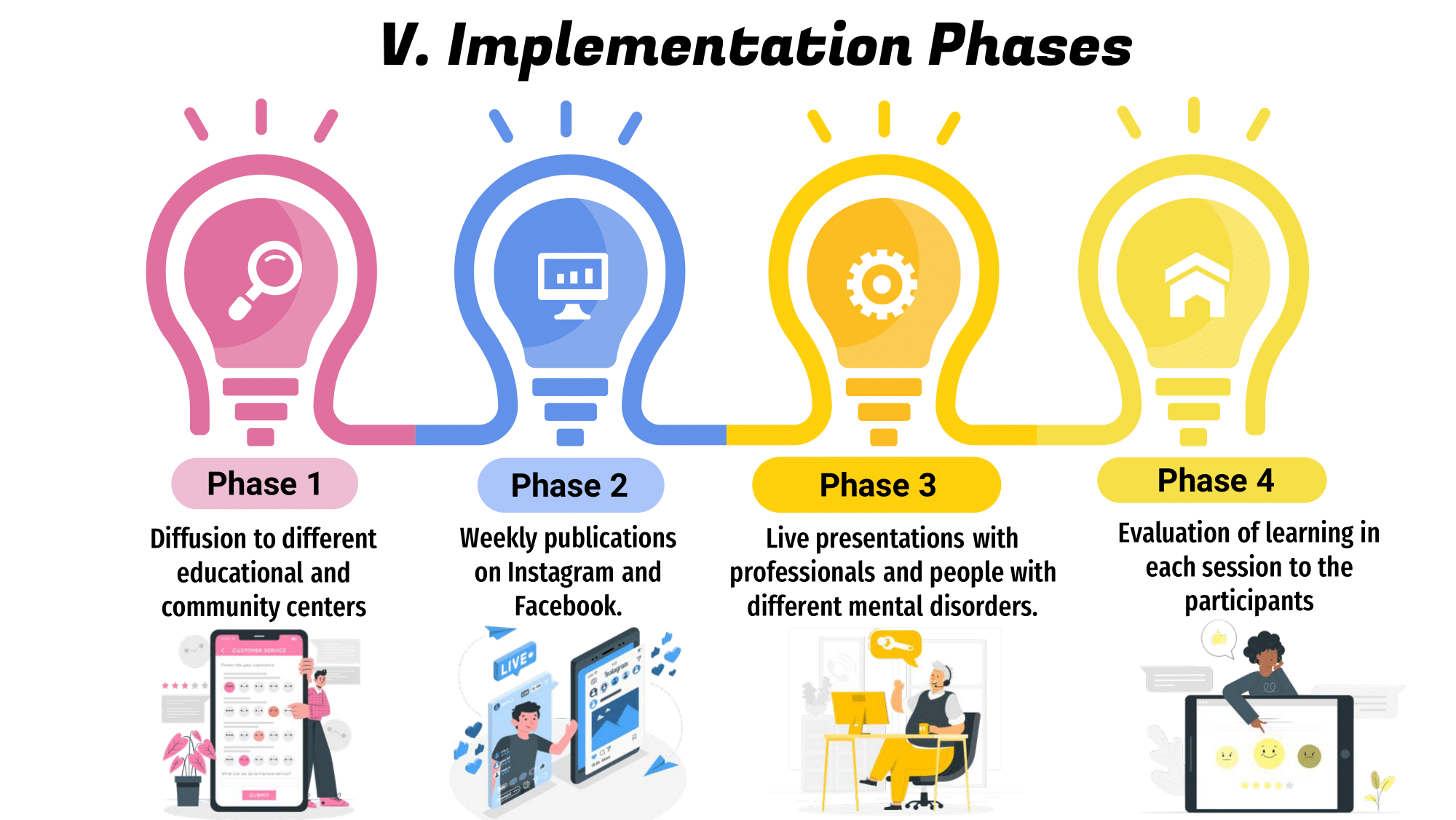


**E**


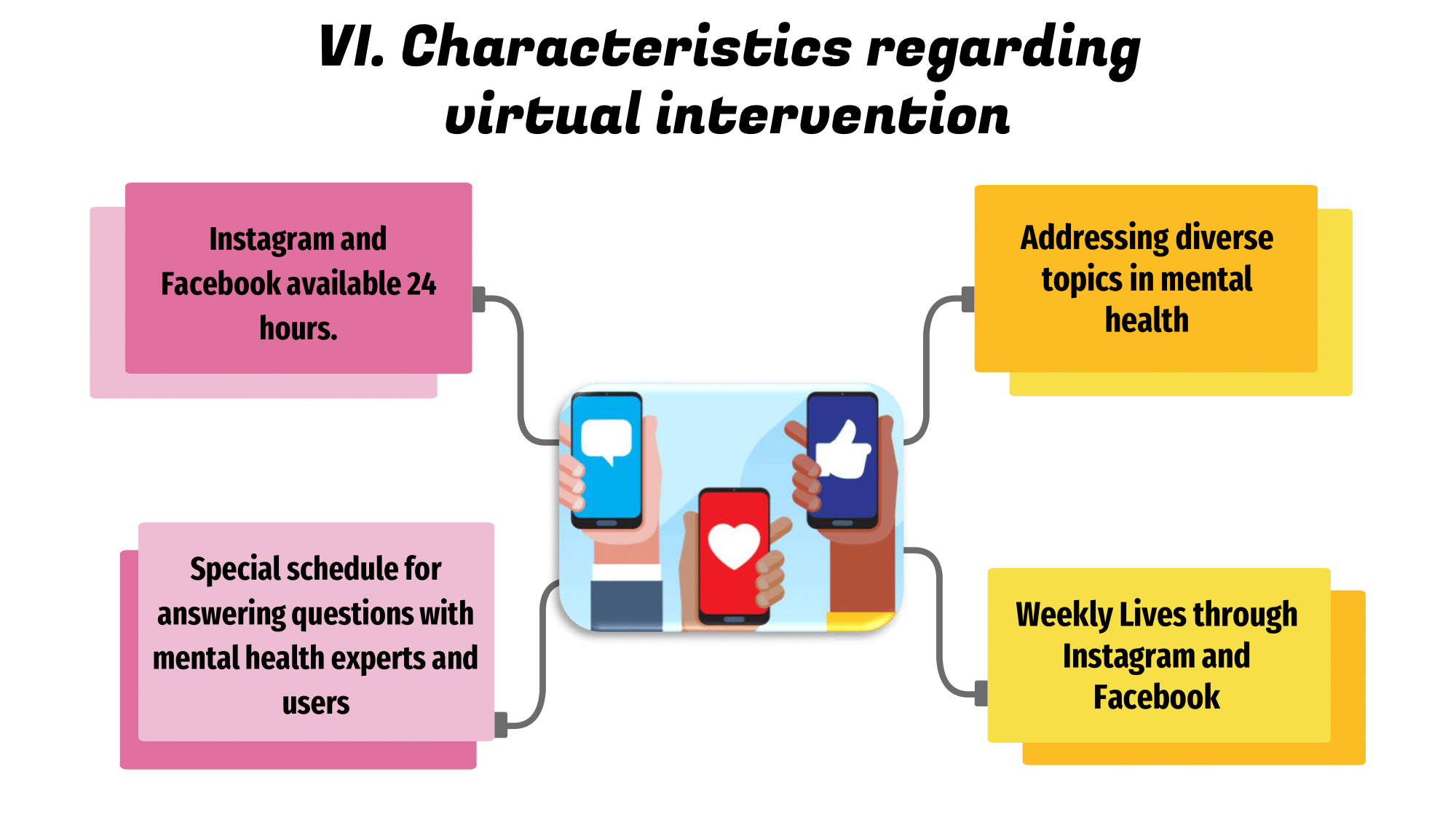


**F**


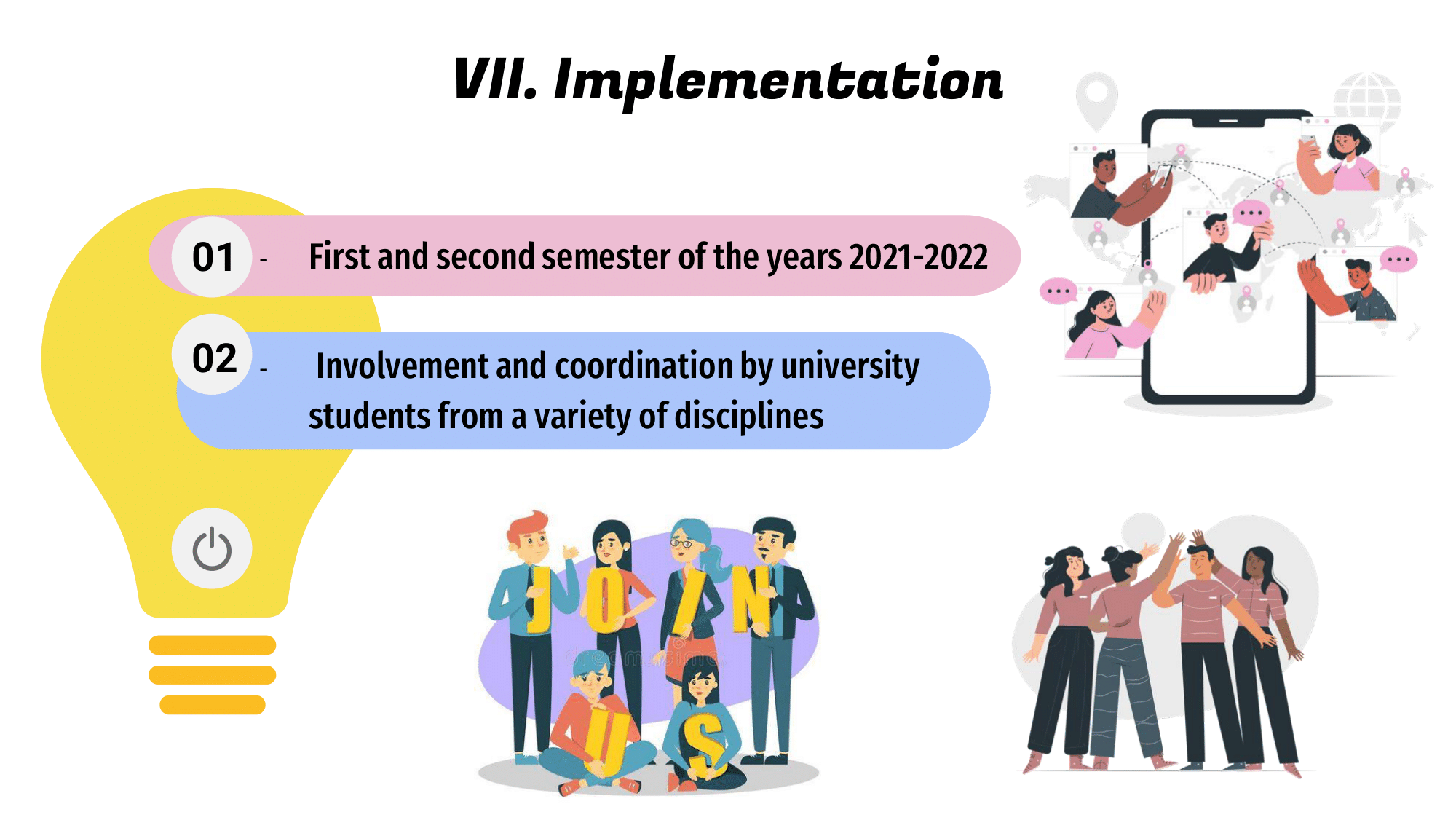


**G**

**Supplementary Figure 1.** A) Intervention project's cover; B) Context of the intervention; C) Introduction and objectives of the intervention; D) Contents of the intervention; E) Project implementation phases; F) Characteristics of the intervention; G) Stage of implementation of the project intervention. Note: English translation of the intervention in reduction of the stigma towards mental illness designed by the university students.
