# Supplementary material for: Controlled Study of the Impact of a Virtual Program to Reduce Stigma Among University Students Toward People With Mental Disorders
Source: Front Psychiatry. 2021 Feb 9;12:632252. doi: 10.3389/fpsyt.2021.632252 (PMC7900522; doi:10.3389/fpsyt.2021.632252)
Supplement: Supplementary file 2 [file Data_Sheet_2.PDF]

Supplementary Material
I. Learning Strategies Assessment Scale

1. Regarding the Project Based Learning Methodology:

	1	2	3	4	5
At what level do you think this methodology can help students in general become more interested in learning about mental health?	◯	◯	◯	◯	◯
At what level do you think this experience can help others improve their understanding of the recovery process in people with serious mental health problems?	◯	◯	◯	◯	◯
At what level do you consider this experience to have been educational, in general, about serious mental health problems?	◯	◯	◯	◯	◯
At what level do you think this experience can make those involved more empathetic towards people with serious mental health problems?	◯	◯	◯	◯	◯
At what level do you think this methodology can be useful to promote the social inclusion of people with serious mental health problems?	◯	◯	◯	◯	◯

2. Regarding Standardized patients and anti-stigma Workshops:

	1	2	3	4	5
At what level do you think this methodology can help students in general become more interested in learning about mental health?					
At what level do you think this experience can help others improve their understanding of the recovery process in people with serious mental health problems?					
At what level do you consider this experience to have been educational, in general, about serious mental health problems?					
At what level do you think this experience can make those involved more empathetic towards people with serious mental health problems?					
At what level do you think this methodology can be useful to promote the social inclusion of people with serious mental health problems?					
What level of immersion or feeling of being in a real context have you felt during the experience?					
What level of entertainment have these simulation activities been for you?					
Would you recommend a friend to experience this programme of simulated interventions?					

3. Regarding E-Contact intervention:

	1	2	3	4	5
At what level do you think this methodology can help students in general become more interested in learning about mental health?					
At what level do you think this experience can help others improve their understanding of the recovery process in people with serious mental health problems?					
At what level do you consider this experience to have been educational, in general, about serious mental health problems?					
At what level do you think this experience can make those involved more empathetic towards people with serious mental health problems?					
At what level do you think this methodology can be useful to promote the social inclusion of people with serious mental health problems?					

4. Regarding Integration of the 3 types of learning strategies:

	1	2	3	4	5
At what level do you think this methodology can help students in general become more interested in learning about mental health?					
At what level do you think this experience can help others improve their understanding of the recovery process in people with serious mental health problems?					
At what level do you consider this experience to have been educational, in general, about serious mental health problems?					
At what level do you think this experience can make those involved more empathetic towards people with serious mental health problems?					
At what level do you think this methodology can be useful to promote the social inclusion of people with serious mental health problems?					
What level of immersion or feeling of being in a real context have you felt during the experience?					
What level of entertainment have these simulation activities been for you?					
Would you recommend a friend/ who experiences these integrated strategies?					

Note: This instrument was designed specifically for the current study.
